# Neuroplastic Changes in Older Adults Performing Cooperative Hand Movements

**DOI:** 10.3389/fnhum.2018.00488

**Published:** 2018-12-13

**Authors:** Lars Michels, Volker Dietz, Alexandra Schättin, Miriam Schrafl-Altermatt

**Affiliations:** ^1^Department of Neuroradiology, University Hospital Zurich, Zürich, Switzerland; ^2^MR-Center, University Children’s Hospital Zurich, Zürich, Switzerland; ^3^Spinal Cord Injury Center, Balgrist University Hospital, Zürich, Switzerland; ^4^Institute of Human Movement Sciences and Sport, ETH Zürich, Zürich, Switzerland; ^5^Neural Control of Movement Laboratory, ETH Zürich, Zürich, Switzerland

**Keywords:** functional magnetic resonance imaging, somatosensory cortex, neural coupling, healthy aging, neuroplastic changes

## Abstract

The aim of this study was to examine whether older adults use the same task-specific brain activation patterns during two different bimanual hand movement tasks as younger adults. Functional magnetic resonance brain imaging was performed in 18 younger (mean age: 30.3 ± 3.6 years) and 11 older adults (62.6 ± 6.8 years) during the execution of cooperative (mimicking opening a bottle) or non-cooperative (bimanual pro-/supination) hand movements. We expected to see a stronger task-specific involvement of the secondary somatosensory cortex (S2) during cooperative hand movements in older compared to younger adults. However, S2 activation was present in both groups during the cooperative task and was only significantly stronger compared to the non-cooperative task in younger adults. In a whole brain-analysis, the contrast between older and younger adults revealed a hyperactivation of the bilateral dorsal premotor cortex (precentral gyrus), right thalamus, right frontal operculum, anterior cingulate cortex, and supplementary motor areas in older adults (*p* < 0.001), with some of them being visible after correcting for age. Age was positively associated with fMRI signal changes in these regions across the whole sample. Older adults showed reduced gray matter volume but not in regions showing task-related fMRI group differences. We also found an increase in functional connectivity between SMA, M1, thalamus, and precentral gyri in older adults. In contrast, younger adults showed hyperconnectivity between S2 and S1. We conclude that older compared to younger adults show age-related functional neuroplastic changes in brain regions involved in motor control and performance.

## Introduction

Aging leads to a decline in movement performance (Eudave et al., 2016; Zapparoli et al., 2016). During hand or foot movements, this is associated with an impaired limb coordination, and an altered brain activation (Heuninckx et al., 2004, 2005; Goble et al., 2010, 2012; Heitger et al., 2013). Using perfusion positron emission tomography, it has been shown that older adults demonstrate a hyperactivity (compared to younger adults) of cortical areas even in simple auditory-cued finger (Calautti et al., 2001) or hand (Hutchinson et al., 2002) movements. According to a functional magnetic resonance imaging (fMRI) study, a general increase in the activation of sensorimotor networks, including areas involved in cognitive monitoring of movement performance and integration of information from the two sides of the body, occurs in older participants who performed isolated flexion and extension of the wrist or ankle (Heuninckx et al., 2005). These findings indicate an age-related shift along a continuum from automatically to more “consciously” controlled movement performance. The observed hyperactivity was suggested to compensate sensorimotor deficits and counteract an age-related decline in motor function (Heuninckx et al., 2005). However, other studies reported age-related cortical hyperactivation using fMRI during bimanual coordination tasks not only in somatosensory cortex but also in the dorsolateral prefrontal cortex, inferior frontal gyrus (IFG), supplementary motor area (SMA), inferior parietal- and cingulate cortex (Coxon et al., 2010; Goble et al., 2010; Maes et al., 2017). Hyperactivity was also seen in older adults in other motor studies (Ward and Frackowiak, 2003; Ward, 2006; Heuninckx et al., 2008; Van Impe et al., 2009, 2011), indicating that this neuroplastic age-related change (i.e., brain’s ability to reorganize itself by forming new neural connections during aging) could be a generic rather than task-dependent process.

Recently, stronger activation of the bilateral secondary somatosensory cortex (S2) during cooperative (mimicking opening a bottle using a hand-made device) compared to non-cooperative hand movements has been described (Dietz et al., 2015). This finding supported the role of S2 being responsible for the integration of sensory information from both hands (Lin and Forss, 2002; Wenderoth et al., 2005), which is specifically required for the movement coordination between the two sides during cooperative hand movements. The task specific neural coupling underlying cooperative hand movements could also be demonstrated by electrophysiological experiments (Schrafl-Altermatt and Dietz, 2014; Dietz et al., 2015). These electrophysiological findings were confirmed to be valid also in older participants (Schrafl-Altermatt and Dietz, 2016a,b).

The aim of this study was to evaluate whether S2 shows age-dependent changes in the fMRI signals during cooperative hand movements as a sign of neuroplastic changes. According to the compensation hypothesis (Goble et al., 2010), it is assumed that S2 becomes more strongly activated in older adults compared to younger participants during cooperative hand movements. Alternatively, hyperactivity in older subjects might become task-independent and could be present in brain regions in and outside of S2, as described in previous studies (Coxon et al., 2010; Goble et al., 2010). We applied two types of analyses to examine the research question: (1) a whole-brain approach to study task and group differences in and outside of S2, and (2) a task-related functional connectivity (FC) approach. The latter approach enables to assess local and distributed connectivity based on the comparison of temporal changes in task-dependent brain activity between groups, as a measure of temporal fMRI signal coherence on the intra- and interhemispheric level.

## Materials and Methods

### Participants

Imaging data of 22 adults was reported in a previous report (Dietz et al., 2015). We selected a younger subsample of the previous study population (*n* = 18) to have a more homogeneously distributed age range (i.e., age differs maximally one standard deviation from the mean age across the group). New data of 21 participants (age ≥ 45 years) was collected. For the final sample size and age ranges, we refer to the results section. All participants were right-handed (Oldfield, 1971). Demographic data was compared between groups using Chi square test or independent Student’s *t*-test. Participants were recruited from the local community (University Hospital Zurich and Balgrist University Hospital) by word of mouth.

### Procedure and Design

This study was approved by the Ethics Committee of the Canton of Zurich and conformed to the standards set by the Declaration of Helsinki. Participants were informed about the purpose of the experiment and all gave written informed consent for their participation. Participants performed two tasks during fMRI. Each task was performed in a separate run using a block design. The full details of the tasks were described elsewhere (Dietz et al., 2015). In brief, participants performed a cooperative hand movement task mimicking the opening and closing of a bottle using a custom-made and hand-held MR compatible device and a bimanual non-cooperative task of pronation and supination with fMRI-compatible dumb-bells. The sequence of fMRI runs (i.e., cooperative and non-cooperative tasks) was randomized across participants and was balanced within each group to minimize order effects, i.e., odd-labeled (1, 3, etc.) participants in each group (young and old adults) started with the cooperative hand movement task, whereas even-labeled participants of each age group started with the non-cooperative hand movement task. For all tasks, a centrally presented green or red rectangle was shown for two seconds alternatively. During each of the 10 blocks, participants had to perform seven supination or “opening the bottle” movements on the green cue and seven pronation or “closing the bottle” movement with the red cue (block duration: 28 s). We visually controlled the performance of movements with a camera installed in the MRI scanner room (movement patterns were not recorded). Each block of active movements was followed by a baseline block, with a centrally presented yellow rectangle (same size as the red and green rectangle) shown for 8–10 s (mean duration 9 s). Participants were instructed to fixate the rectangles presented on the screen, which was visible via the mirror in the head coil. The occurrence of eye movements was thereby minimized. Ten active and ten resting blocks were presented for each task. The synchronization between the fMRI clock and the temporal onset of the visual cues was controlled by Presentation^1^. Participants were familiarized with the movements before starting the experiment and showed no difficulty performing the movements smoothly and correctly. The two tasks were comparably simple regarding execution.

### Data Recording

Functional MRI images were acquired on a 3-T Philips Ingenia whole-body MRI system (Philips Medical System, Best, Netherlands) at the University Hospital Zurich equipped with an 8-channel head coil. Cortical activation was examined in 181 scans per run using 30 transverse slices in oblique orientation. The blood oxygen level-dependent imaging parameters (echo time = 35 ms, repetition time = 2 s, field of view = 220 mm × 220 mm, voxel size: 2.75 mm × 2.75 mm × 4 mm, reconstructed voxel size: 1.72 mm × 1.72 mm × 4 mm, flip angle = 78°) were employed by the application of the sensitivity encoded (SENSE, factor 1.8) single-shot echo planar imaging (EPI) technique allowing interleaved order of slice acquisition. The use of SENSE imaging shortens the readout trains in single-shot EPI, reduces the susceptibility to artifacts, and improves spatial resolution (Boujraf et al., 2009). Four dummy scans were acquired at the beginning of each run and discarded to establish a steady state in T1 relaxation for all functional scans.

### Data Analysis fMRI

Post-processing was identical to the steps described in our previous study (Dietz et al., 2015). All fMRI images were post-processed with MATLAB 7.9 (Mathworks Inc., Natick, MA, United States) and the Statistical Parametric Mapping software (SPM8, Wellcome Trust Centre for Neuroimaging, London, United Kingdom). The post-processing steps included realignment (head motion correction using three translation and three rotation values), normalization to the EPI template provided by the Montreal Neurological Institute (MNI brain, McGill University, Montreal, Canada), re-slicing to 2 mm × 2 mm × 2 mm voxel size (i.e., to match the EPI template resolution), and spatial smoothing using 8-mm full-width at half-maximum Gaussian kernel. An autoregressive model of the first order was used to account for serial correlations. High-pass filtering with a standard 128 s cut-off eliminated slow signal drifts. A standard hemodynamic response function was used for convolution of the model regressors. First-level analyses were conducted using a voxel-wise generalized linear model (GLM), which reflects a flexible generalization of an ordinary/simple linear regression (Friston et al., 1995). We included six head motion parameters as regressors of no interest (covariate) in the first-level single-subject analysis to minimize signal variance explained by head motion despite the fact that the groups did not differ with respect to any of the parameters (*p* > 0.05 for all rotation and translation values). For the group analysis, each task was entered as separate regressor into 2 × 2 repeated-measures ANOVA containing the factors *task* (cooperative and non-cooperative hand movements) and *group* (younger and older adults). We first tested for a main effect of age and task as well as for group × task interaction effects. In case of a significant effect (*p* < 0.05, cluster-corrected), we performed *post hoc* tests. For within-group comparisons, we applied comparisons using paired *t*-tests (with the same statistical threshold). Unpaired *t*-tests were performed for between-group contrasts. The cluster extent threshold was obtained by simulating whole-brain fMRI activation using cortex custom software written in MATLAB (Slotnick et al., 2003). In a single simulation, by modeling the entire functional image matrix, assuming a type I error voxel activation probability of 0.001, and smoothing the activation map by convolution with a 3-dimensional 8 mm FWHM Gaussian kernel, the size of each contiguous cluster of voxels was determined. After 10,000 simulations, the probability of each cluster size was determined and the cluster extent that yielded *p* < 0.05, i.e., 29 contiguous resampled voxels, was selected for use in voxel extent thresholding.

### Additional Between- and Within Group Differences and Correlations

Additional between-group comparisons were performed for each task using age as covariate in the model to account for the age range in each group. Further, we performed a ROI-based analysis in brain regions showing a significant group difference by calculating Pearson correlations between age and brain activity (beta weights) across younger and older adults. ROI-related activity was extracted using a sphere (6 mm diameter) around the MNI peak coordinate. Within-group correlations were calculated as Spearman’s correlations. Polynomial regressions of second order were fitted to overall FC × age and beta-weights × age data. The highest points of the parabola were calculated to estimate at what age peak values are reached. All results are reported at *p* < 0.05.

### Voxel-Based Morphometry

We applied voxel based morphometry (VBM), which is implemented in SPM8^2^, to examine structural group differences. The VBM analysis followed standard steps and was performed using the T1-weighted 3D data to obtain gray matter volumes (GMV) for eight of 18 younger adults (we did not record T1 images for the other younger adults due to time constraints) and for all eleven older adults. The MRI images were first segmented into GM, white matter (WM), and cerebrospinal fluid (CSF) and images were bias corrected using a unified model inversion algorithm (Ashburner, 2007). For each participant, this resulted in three tissue images in the same space as the original anatomical image, in which each voxel was assigned a probability of being GM, WM, and CSF, respectively. The GM and WM segments were then warped into a reference space [MNI average brain template, Evans et al. (1992)] using a diffeomorphic non-linear image registration tool (using DARTEL) (Ashburner, 2007). Finally, the GM volumes were scaled with the Jacobian determinants estimated by the registration step (i.e., “modulation”) in order to preserve the local tissue volumes and resulting images were smoothed using an isotropic Gaussian kernel with 8 mm full width at half maximum. We performed separate group comparisons using GM controlling for total intracranial volume. Results were reported at *p* < 0.05 (cluster-corrected, *k* > 29 voxels). We also compared GMV and total intracranial volume (i.e., the sum of GM, WM, and CSF) between groups.

### FC

We investigated FC related to spontaneous background activity (Fox et al., 2006; Fox and Raichle, 2007; Norman-Haignere et al., 2012) during the cooperative hand movement task to test for intra- and interhemispheric FC differences between groups. FC was analyzed using the CONN toolbox, version 17e (Whitfield-Gabrieli and Nieto-Castanon, 2012). We used the default steps suggested for resting-state fMRI connectivity analysis. First, outlier detection (using ARtifact detection Tools) and scrubbing (Power et al., 2012) was performed additionally to the post-processing steps described in the Section “Data Analysis fMRI” (realignment, normalization, spatial smoothing, high-pass filtering). Next, data were band-pass filtered (0.01–0.1 Hz) as recommended for FC analysis (Biswal et al., 1995). WM and CSF signals were used as covariates of no interest in addition to the six motion parameters to reduce variance unlikely to reflect neuronal activity. Only the WM and CSF signals were removed to avoid any bias introduced by removing the global signal (i.e., GM). This denoising step has been shown to “normalize” the distribution of voxel-to-voxel connectivity values as effectively as including the global signal as a covariate of no interest but without the potential problems described for the latter method (Behzadi et al., 2007; Murphy et al., 2009). Additionally, linear detrending was performed during the denoising step. After the denoising step, the distribution of voxel-to-voxel FC was visualized for each step.

We compared FC in regions involved in motor and sensory processing. Regions of interests (ROIs) were the midline primary motor cortex (M1), bilateral postcentral gyri (primary somatosensory cortex, S1), SMA, S2, thalamus, and precentral gyrus (PCG). All ROIs, except for S2, were extracted from the WFU_pickatlas (Maldjian et al., 2003). The S2 ROI was chosen identical to the one described in our previous publication (Dietz et al., 2015), as the WFU_pickatlas does not comprise a single area for S2.

Group differences were investigated by regression analyses. The *t*-values were calculated as *t* = b1/SE (with b1 = slope of the sample regression line, SE = standard error of the slope). All results are reported at *p* < 0.05 (two sided) corrected for multiple comparisons using false discovery rate (FDR) correction.

## Results

### General

Data from seven older adults had to be removed due to excessive head movements (>3 mm translation) during one or more fMRI runs. In addition, we only included participants with an age ≥53 years narrowing the age range in this group. Hence, the final sample consisted of 18 young adults (mean age: 30.3 ± 3.6 years, range 24–36 years) and 11 older adults (mean age: 62.6 ± 6.8 years, range 53–73 years, 7/11 > 60 years). Groups differed significantly in age (*p* < 0.001 unpaired two-sample *t*-test) but not in gender (*p* > 0.05, Chi-square test).

### FMRI

**FIGURE 1:**
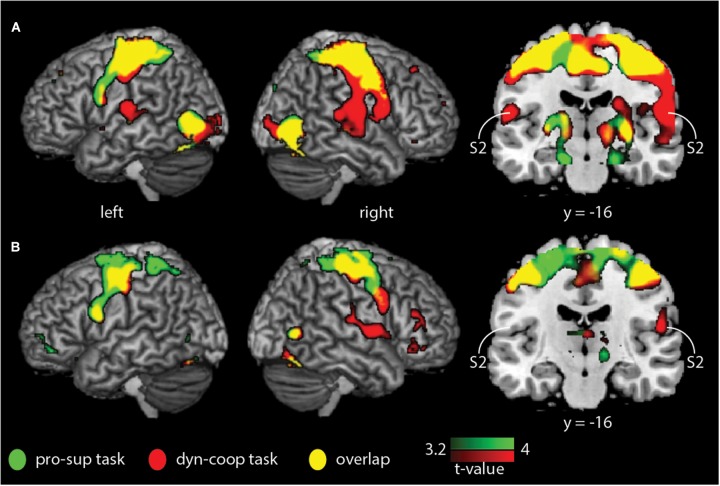
Whole-brain within-group analysis indicating BOLD signal changes for younger and older adults for the non-cooperative (green) and the cooperative (red) hand movement tasks. **(A)** Younger adults; **(B)** older adults. Results are shown at *p* < 0.05 (cluster-corrected). S2, secondary somatosensory cortex.

**FIGURE 2:**
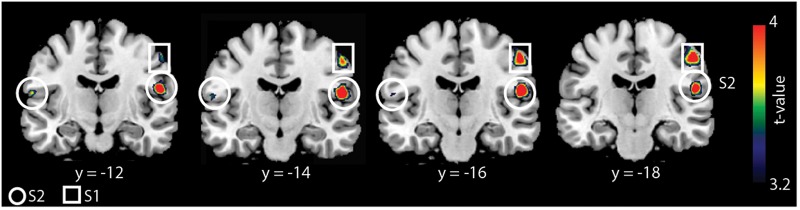
Whole-brain analysis for younger adults for the contrast “cooperative versus non-cooperative task.” Results are shown at *p* < 0.05 (cluster-corrected). BOLD signal changes are seen in the bilateral S2 and right S1.

We found a main effect of group and task (*F* = 12.11, *p* < 0.05, cluster-corrected) but not a group × task interaction. Whole-brain activation maps for both groups and tasks are shown in Figure 1. Activation looks comparable between groups, yet it appears that S2 was activated less strongly in older compared to younger adults. As we found significant main effects of group and task, we then applied different statistical comparisons on the *post hoc* level. First, we compared within group-differences comparing the two tasks. Then, we performed (for each task) statistical comparisons between groups. As reported in our previous study (Dietz et al., 2015), we found stronger BOLD signal changes for the contrast “cooperative versus non-cooperative hand movements” in a smaller sample of younger adults (Figure 2 and Table 1) in bilateral S2 and right S1. However, none of the other within-group comparisons revealed a significant difference.

**Table 1:** Summary of BOLD signal differences for the contrast “cooperative hand movement task versus non-cooperative hand movement task” for younger adults.

Brain region	Hemisphere	Peak MNI coordinate	Cluster size	Peak *t*-value
S2	Left	-60, -12, 16	30	3.25

S2	Right	54, -14, 18	99	3.87

S1	Right	52, -18, 46	141	3.34


**FIGURE 3:**
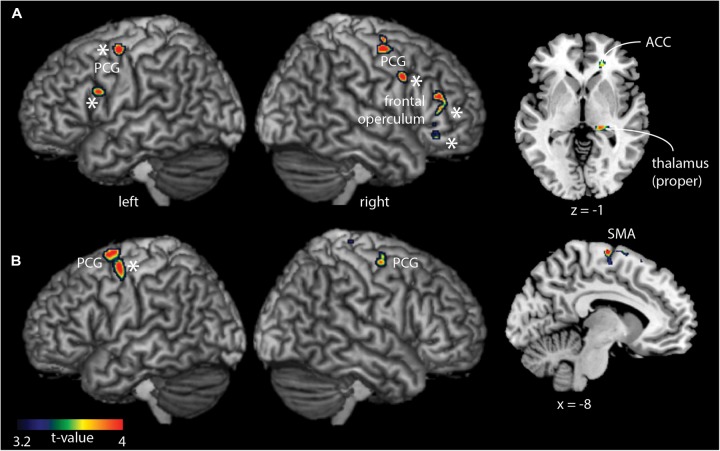
Whole-brain between-group fMRI analysis for the cooperative task **(A)** and non-cooperative task **(B)**. Older adults demonstrated significantly (*p* < 0.05, cluster-corrected) stronger fMRI signal changes compared to young adults in precentral and frontal brain regions in addition to the thalamus (cooperative task only) and the supplementary motor area (SMA, non-cooperative task). ^∗^Regions that survive a correction for age.

Comparing groups for each task, showed hyperactivity exclusively in the group of older participants (Figure 3 and Table 2). The hyperactivity was found in the bilateral PCG, thalamus, IFG/frontal operculum (FO), and anterior cingulate cortex (ACC) for the cooperative movement task, and in the PCG and SMA for the non-cooperative movement task. Most of the reported regions survive as correction for age (indicated by ^∗^).

**Table 2:** Summary of BOLD signal differences for the contrast “older adults – younger adults” during the performance of the non-cooperative task and the cooperative hand movement task.

Brain region	Task	Hemisphere	Peak MNI coordinate	Cluster size		Peak *t*-value
	***Non-cooperative***					
Precentral gyrus		Left	-40, -4, 58		275	4.28
		Right	30, -8, 58		94	4.16
Supplementary motor area		Left	-8, -144, 70		36	5.04
					
	***Cooperative***					
Precentral gyrus		Right	42, -8, 56		58	3.77
		Left	-42, -6, 58		44	3.26
Thalamus		Right	8, -24, 12		51	3.67
Anterior cingulate cortex		Right	20, 38, -2		82	3.66
Inferior frontal gyrus (frontal operculum)		Right	34, 32, 14		517	3.71


### FC

**FIGURE 4:**
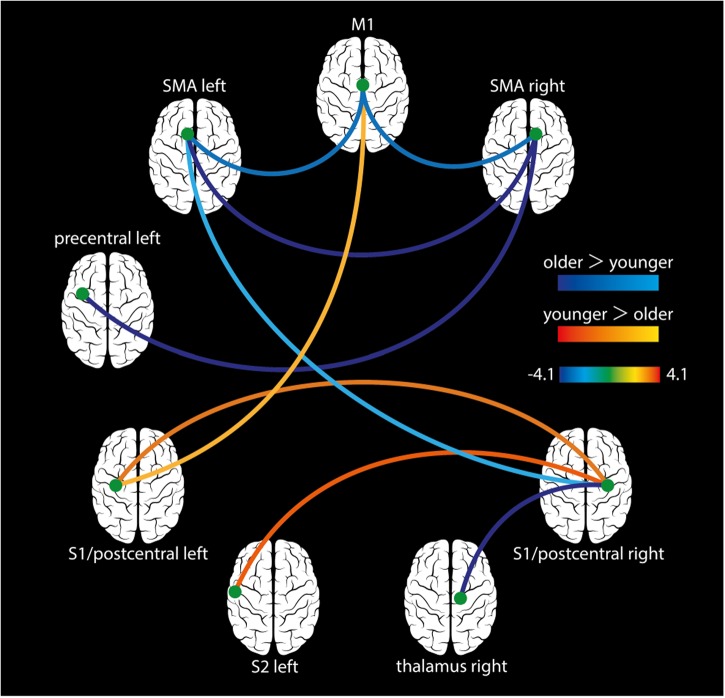
Task-based FC analysis. During the cooperative hand movement task, older adults showed hyperconnectivity compared to younger adults for the following connections: left SMA – right SMA, left SMA – M1, right SMA – M1, left SMA – right S1, right SMA – left PCG, and right S1 – right thalamus. Younger adults showed higher connectivity between the left S1 – right S1, left S2 – right S1, and left S1 – M1. All results are shown on *p* < 0.05 (FDR-corrected).

All participants showed normally distributed data after denoising and were therefore included for further analyses. We found significant hyperconnectivity in older adults for the following functional connections: left SMA – right SMA, left SMA – M1, right SMA – M1, left SMA – right S1, left PCG – right SMA, and right S1 – right thalamus. In contrast, younger adults showed significant hyperconnectivity for theses connections: left S1 – right S1, left S2 – right S1, and left S1 – M1 (Figure 4 and Table 4).

### Age-Dependent Brain Activation

**FIGURE 5:**
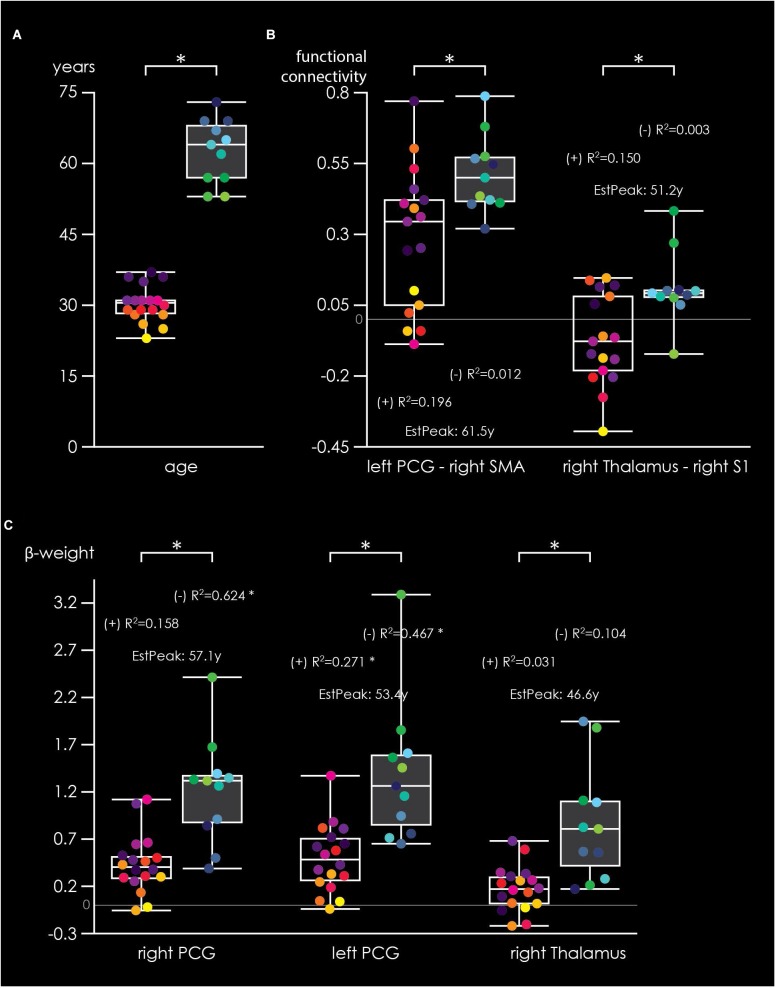
Box plots illustrating age-related effects during the cooperative task. **(A)** Age distribution of younger (black boxes, yellow-purple subject dots) and older (gray boxes, green-blue subject dots) adults. **(B)** FC for left precentral gyrus (PCG) – right SMA and for right thalamus – right primary somatosensory area. **(C)** Beta-weight distribution for three regions of interest (right PCG, left PCG, and right thalamus). *R*^2^-values represent within-group Pearson coefficients for FC/beta-weight × age correlations, with (+) indicating a positive and (-) a negative correlation. “EstPeak” indicate the estimated age in years when the peak of FC/beta-weight is reached. ^∗^Indicate significant (*p* < 0.05) between-group differences or within-group correlations.

Figure 5 shows differences in brain activation and FC between the two age groups for the cooperative hand movement task. Figure 5A shows the age in years of the included participants. As shown in 5B, FC was significantly stronger in older adults compared to younger adults for left PCG – right SMA as well as for right thalamus – right S1. Within the young group, non-significant positive correlations were found for both FCs while no correlation was found within older adults. The estimated peak-connectivity was 61.5 years for left PCG – right SMA and 51.2 years for right thalamus – right S1. Figure 5C shows significant differences in beta-weights between younger and older adults for right PCG, left PCG as well as right thalamus. In all three regions, activity was stronger in older adults. Within group analysis, however, revealed positive correlations only within younger adults (significant for left PCG) but negative correlation within the older group (significant for right and left PCG). Estimated peak-activity amounted to 57.1 years for right PCG, 53.4 years for left PCG and 46.6 years for right thalamus, respectively. Older adults also showed higher beta-weights during the non-cooperative task (Supplementary Figure S1). However, no within-group correlations were found and no peak-age could be estimated. The task × age fMRI analysis indicates similar group differences as shown by the 2 × 2 ANOVA, although the effects were most pronounced in the IFG/OP (Supplementary Figure S2).

### Brain Structure

We observed significantly higher GMV in younger adults in several brain regions (Table 3). However, none of these changes were present in brain regions activated in the study tasks. Younger adults showed higher global GMV (*p* = 0.03) and total intracranial volume (*p* = 0.02) compared to older adults.

## Discussion

In this study, we examined differences in the brain activation pattern between younger and older healthy adults during cooperative and non-cooperative bimanual movements. According to the compensation hypothesis (Goble et al., 2010) and based on previous results (Dietz et al., 2015), we assumed enhanced task-specific activation of the S2 cortical areas during cooperative hand movements in all participants and an increase of S2 activation in older participants. Both groups showed S2 activation during cooperative hand movements, the contrast to non-cooperative movements was, however, only significant in the younger group. The activation (of S2) appeared visually to be even less strong in older adults (see Figure 1), but the group difference (for each task) was not significant.

The novel finding was that older adults demonstrated spatially widespread hyperactivity in the PCG, thalamus, ACC, IFG/FO (cooperative task), and SMA (non-cooperative task) compared to younger adults, but not in S2. These findings suggest that older adults use a more general, task-unspecific brain activation for the performance of bimanual movements. Importantly, these results are in line with the compensation hypothesis, with the exception of S2 not showing hyperactivity in older adults compared to young adults. The latter could indicate that compensatory changes (i.e., hyperactivity) in S2 might occur later in life or might depend on task difficulty. Our results most likely indicate that the specific function of S2 in the group of younger adults during cooperative hand movements is taken over in older adults by other brain areas showing hyperactivity. Our results – related to S2 – might be in conflict to the study of Heuninckx et al. (2005), as these authors found hyperactivity not only in similar regions found in our study but also in S2. Yet, the group of older adults was older (64.8 years of age; range, 62–71 years versus 62.6 years, range 53 – 73 years), indicating that age (but also the task type) might be a critical factor for observing hyperactivity in older adults in bimanual coordinating tasks, particularly in S2.

Our findings extend a previous study reporting that older adults rely on supplementary circuitries which are important in cognitive operations (Reuter-Lorenz and Park, 2014) as we found activation outside of sensorimotor regions (M1 or S1/S2) but in areas involved in motor control and preparation.

It has not definitively been determined whether changes in the brain activation pattern in older adults during movement performance are compensatory and thus reflect effectivity (for review see Ward, 2006). Studies on movement control of older participants showed an over-activity in more distributed neural networks. This was suggested to mirror the maintenance of a sufficient level of performance (Mattay et al., 2002; Wu and Hallett, 2005) supporting the compensation hypothesis. Other studies using simple motor tasks, i.e., tasks with comparable motor performance in younger and older participants, such as wrist or ankle movements (Heuninckx et al., 2005), isometric hand grip (Ward and Frackowiak, 2003) or finger tapping (Calautti et al., 2001) showed a general increase in the brain activation pattern in older compared to younger participants.

Cooperative hand movements are efficiently performed in both younger and older participants using S2 and other cortical areas for the neural coupling of the two hands. For S1 and S2 it is known that they integrate somatosensory signals from the two sides of the body (Hari et al., 1998; Disbrow et al., 2001; Lin and Forss, 2002; Wenderoth et al., 2005; Tamè et al., 2015a,b). Based on our observations made in older participants, coordination between the two hands becomes also more important for the successful performance of various bimanual movement tasks. In fact, no difference between cooperative and non-cooperative movements were present in the older subject group and a similar pattern of activated regions was shown for both movement tasks. The additionally activated areas (e.g., PCG and SMA) in older adults are known to be involved in motor control, such as planning and execution of movements (for review see Nachev et al., 2008), as well as in the spatial and sensory guidance of movements (Roland et al., 1980a,b). Our findings of stronger activation in the SMA in older adults are in line to another fMRI study that demonstrated higher SMA activity for wrist and ankle movements in older adults compared to younger adults (Heuninckx et al., 2005).

The involvement of the PCG and of the SMA in the control of bimanual movements is in so far plausible that these cortical areas follow the age-related shift to a more controlled movement performance, independently of the type of bimanual task. An interesting finding in the older participants was the specific activation of the thalamus, which has connections to the PCG, seen only in the contrast older-younger during the cooperative task. The thalamus is known to be involved in motor imagery (Muller et al., 2013). This observation might suggest that older adults rely more on visualization/imagery during more complex movement performance. Our findings are not in line with the notion that there is shift from subcortical to cortical activation with aging (for a review see Maes et al., 2017). Yet, our sample of older adults had a mean age of about 63 years, indicating that subcortical regions are still required for bimanual hand coordination.

It is evident that not all sensorimotor regions follow a pattern of increased activation with increasing age. For example, a greater contralateral activation of S1 and PCG was found in younger adults, whereas SMA had significantly higher activation in older adults during a repetitive motor task (Hutchinson et al., 2002). Furthermore, the ipsilateral sensorimotor cortex was stronger activated in older adults during complex (index) finger motor tasks of both hands, pointing toward compensatory recruitment of motor cortical units in this subject group. The bimanual tasks studied here were easy to perform but, nevertheless a stronger activation was observed in older adults. We hence conclude that age-related increases in brain activation are not solely due to task-difficulty but can also be task-independent.

It has been described that handedness significantly influences bimanual coordination on the behavioral and physiological (shown by electroencephalography, EEG) level (Kourtis et al., 2014). However, as all our participants were right-handed, we conclude that none of the between group differences are influenced by handedness.

### Age-Dependent Brain Activation

In general, fMRI and positron emission tomography studies in aging have demonstrated regional hyperactivation in the motor but also in cognitive and perceptual circuits (Grady et al., 1994; Cabeza, 2001; Calautti et al., 2001; Cabeza et al., 2002; Logan et al., 2002; Mattay et al., 2002; Ward and Frackowiak, 2003; Park et al., 2004; Naccarato et al., 2006; Riecker et al., 2006; Heuninckx et al., 2008; Van Impe et al., 2011; Sala-Llonch et al., 2015). Two hypotheses (and modifications hereof) have been proposed to explain such hyperactivations with aging: dedifferentiation and compensation. The dedifferentiation hypothesis stresses a decrease in functional specificity during task performance in older adults (Grady et al., 1994; Logan et al., 2002; Park et al., 2004; Sala-Llonch et al., 2015), most likely due to reduced inhibitory control. The compensation hypothesis formulates that the additional recruitment of brain regions is a compensatory mechanism for functional and/or structural deficits in particular brain regions (Calautti et al., 2001; Mattay et al., 2002; Naccarato et al., 2006; Heuninckx et al., 2008). In fact, the compensation hypothesis is not applicable in case hyperactivations are behaviorally not beneficial anymore due to higher task demand (Reuter-Lorenz and Cappell, 2008; Schneider-Garces et al., 2010; Grady, 2012). However, as the task demand was low in our study, we think that this is not a valid hypothesis to explain our results.

It has also been shown in EEG studies that aging leads to elevated alpha and beta desynchronization and lower functional hemispheric asymmetry in older adults (Labyt et al., 2003, 2004, 2006; Vallesi and Stuss, 2010; Rossiter et al., 2014; Schmiedt-Fehr et al., 2016). During bimanual finger tapping coordination task, it was shown in an EEG study that older adults showed increased desynchronization in the alpha and beta band, being present over parietal (alpha) and sensorimotor (beta) brain areas (Blais et al., 2014). Our results support the notion of lower unilateral task-related activation, as we found group differences for both tasks bilaterally at least in the PCG (see Figures 1, 3). Our findings might indicate a loss of neuronal selectivity for a particular motor task but with preserved motor performance. In contrast, we did not observe group differences in the parietal cortex for both tasks. This might be explained by the fact that our task was easy to perform and did not, as in Blais et al. (2014), require intermediate phase coordination. In younger adults, a positive relationship between age and fMRI signal strength was found for the cooperative task within each of the three examined ROIs (Figure 5). However, in older adults (showing hyperactivity in these ROIs), there was a significant inverse association to age in the PCG. This indicates that at a certain age hyperactivity tends to decrease again in some brain regions, while it stabilizes in others. A similar, though not statistically significant, effect was found for the FC in two of these three ROIs. FC seems to become strengthened with increasing age, which is in line with previous research (Heitger et al., 2013). At a certain point, the increase seems to stop at the FC level. The peak age of FC is higher than the peak age estimated for the fMRI signal strength. This might indicate that there is an alteration of increasing activity and strengthening FC in order to maintain motor performance with increasing age. For example, inclusion of an older group of participants might have revealed a positive relation between age and hyperactivity again. During the non-cooperative task, no initial increase followed by decrease in older adults was observed. Only hyperactivity in older adults compared to younger adults was shown during this task. The non-cooperative task might not be as challenging as the cooperative movement and motor performance may be maintainable by just increasing activity without strengthening FC.

Using the whole sample, we found similar brain regions (spatially more pronounced at the IFG/FO) in an fMRI × age correlation (Supplementary Figure S1). In addition, the results reported in Figure 3 (based on the 2 × 2 ANOVA), survive for each group comparison for each task if corrected for age. Taken together, these findings suggest (1) the presence of task-independent age-related neuroplastic changes (increases) to maintain motor task performance and (2) that these fMRI effects can be reliably detected using different statistical approaches, i.e., using an ANOVA with and without age correction or an age × fMRI task activation correlation across the whole sample.

It has been also shown in younger adults that there is a functional dissociation with respect to the internal versus external control of bimanual hand movements (Debaere et al., 2003). The PCG and thalamus (and other brain regions) are involved when movement were guided by visual feedback. In contrast, the SMA (and other brain regions) are predominately involved when movements were internally generated. Based on our findings, we found that visually guided movements (as present for both tasks) activated the PCG and thalamus. A novel finding of our study is that this activation was more pronounced in older adults and additionally involved the SMA (non-cooperative task). We hence suggest that it is not only the type of movement control that could trigger activation in these brain regions but also age. However, as we did not test internal bimanual hand movements, we cannot comment on whether fMRI signal responses would differ in older (and younger) adults between internal versus external control of bimanual hand movements.

### Relationship Between Structure and Function

Older adults showed GMV volume decline and also lower global GMV and total intracranial volume. Importantly, none of the regions showing functional group differences demonstrated an altered GMV (see Table 3). We hence conclude that the observed functional differences were not driven by differences in GMV, i.e., any form of atrophy. This result was expected as our group of older adults was on average 62.8 years and included only 5 (out of 11) participants above 65 years.

**Table 3:** Gray matter volumes (GMV) differences between older adults and younger adults.

Region	Hemisphere	Cluster size	Peak *t*-value	MNI coordinate		
				
				x	y	z
Planum temporale	Left	1043	7.35	-56	-36	14
Middle cingulate gyrus	Left	3650	7.31	-1	-5	21
Brain stem	Left	1812	6.71	-2	-15	-24
Anterior insula	Right	869	6.64	45	14	-2
Cuneus	Right	3655	6.07	1	-75	29
Superior parietal lobe	Left	229	5.91	-27	-65	45
Superior parietal lobe	Right	1717	5.82	35	-57	50
Medial prefrontal cortex	Left	2315	5.75	-5	42	-20
Postcentral gyrus	Left	157	5.53	-57	-20	38


*Younger adults demonstrated higher GMV. All results are reported at p < 0.05 (cluster-corrected).*

**Table 4:** Summary of group differences in task-based FC.

Comparison	Connections
Older adults > Younger adults	Left SMA – right SMA
	Left SMA – M1
	Right SMA – M1
	Left SMA – right S1
	Right S1 – right thalamus
	Left PCG – right SMA
Younger adults > Older adults	Left S1 – right S1
	Left S1 – right S1
	Left S1 – M1


*All results are reported at p < 0.05 (FDR corrected).*

### FC

An adequate motor coordination of bimanual movements in older adults may not only be achieved by an additional neural recruitment but also by aging-related changes of functional relationships between brain regions (Heitger et al., 2013). It has been shown using fMRI that functional connectivity across various brain regions or networks is reduced in older adults (Sala-Llonch et al., 2015). However, aging during cognitive tasks can lead to both increased and decreased FC (Sala-Llonch et al., 2015). Only few studies have examined task-based FC using EEG in the elderly (Vecchio et al., 2014). Vecchio et al. (2014) reported shorter normalized characteristic path length than younger adults in the alpha band, suggesting disturbed efficiency in communication between distant brain regions. Heitger et al. (2013) examined task-related fMRI FC in younger and older adults during continuous and difficulty matched (across groups) in-phase and anti-phase wrist movements. The authors found tighter functional communication, as measured by a shortened communication path length, in addition to stronger task fMRI activity in older adults. Notably, higher FC was present in older adults for both tasks, probably indicating neuroplastic adaptations to resist the loss of coordinative stability.

In the present study, the task-based (cooperative hand movement task) FC analysis indicates that the SMA (bilaterally), M1, right thalamus, right PCG, and postcentral gyri were hyperconnected in older compared to younger adults. In contrast, S1 and S2 were hyperconnected in younger adults. These results suggest that age-related effects were not only produced by group differences in the fMRI signal amplitude but also by coherence differences between the two brain regions. We conclude that stronger interhemispheric interactions, mediated by the corpus callosum, is required for bimanual coordination in older adults. The enhanced connectivity was present in areas responsible for motor preparation and execution (PCG and M1) as well as for sensory processing (thalamus). In contrast, younger adults rely more on interhemispheric integration of brain areas integrating sensory inputs from both body sites (i.e., S1 and S2). It has been shown that anatomical connections underlie FC networks (Damoiseaux and Greicius, 2009; Greicius et al., 2009), although this is still a matter of debate (Honey et al., 2009, 2010). For example, full functional coherence (fMRI signal amplitudes are 100% aligned in phase between two or more brain regions) does not implicate that these brain regions are monosynaptically connected. It is also known that task instruction (Norman-Haignere et al., 2012) and task performance (Sepulcre et al., 2010) can modulate FC. The function of ongoing activity is intimately related to attention and task preparation (here a motor responses). Even though the strength of FC is constrained by structural connectivity, it is modulated by mental states and current context, such that intrinsic activity “constitutes the brain’s internal context for processing external information and generating behavior” (Kenet et al., 2003; Fontanini and Katz, 2008; Sadaghiani et al., 2010).

We hence interpret our FC findings in younger and older adults as representing stronger intrinsic task-related background activity in the described brain regions. Our study extends the notion that hyperactivation in older adults mirrors the maintenance of a sufficient level of performance (Mattay et al., 2002; Wu and Hallett, 2005) supporting the compensation hypothesis, as we found not only hyperactivation but also hyperconnectivity in older adults. WM signal (as well as CSF signal) was removed in the analysis to minimize the influence of physiological “noise”. Hence, all reported FC results are unlikely to be mediated by differences in the WM signal.

### Task Difficulty

Our findings suggest increasingly task-independent activations of sensorimotor cortical areas as a function of age during the performance of various bimanual hand movements. As our task was not designed to examine behavioral parameters such as accuracy or speed, we could not evaluate whether stronger brain activation was associated with task effort. However, both tasks were rather easy and comparable in difficulty, despite the higher complexity of the cooperative task. Visual control of movement performance by an examiner confirmed stable, smooth and correct performance of both tasks by all participants. Increasing speed and testing accuracy during cooperative movements might be an interesting approach in the future as it has been shown that an increase in brain activation – especially in older adults – is positively correlated to task difficulty (Heuninckx et al., 2005). However, having a constant and easy task difficulty has also a major advantage: any of the observed group differences are unlikely to be “biased” by behavioral differences between groups but rather reflect age-related functional changes. In this context, it is known that the control of amplitude and direction can contribute to bimanual coordination (Pan and Van Gemmert, 2018). In our study, both tasks differed with respect to movement direction and amplitude (i.e., different movement directions comparing tasks). Potentially, this could explain some of the observed activation differences comparing cooperative to non-cooperative task at least in younger adults. However, examining the spatial relationship between hand movements when their amplitude and direction is altered, was not systematically tested in this study. Hence, we don’t know whether a different spatial relationship between hand movements and varying movement amplitude or direction would have an impact on the brain activation in younger and/or older adults.

### Limitations of the Study

A limitation of this study is that our group of “older participants” was not really old compared to other studies (e.g., Heuninckx et al., 2005), as we also included few participants below 60 years. However, we observed a similar pattern of hyperactivity in older adults without and with correcting for age (i.e., correction for the distribution of age within each group) and for an age × fMRI signal correlation (across all participants), indicating that older adults demonstrate hyperactivity irrespectively of the performed statistical analysis. The latter finding indicates that the examined age range was wide enough to capture an age-related shift along a continuum from automatically to more “consciously” controlled movement performance in older adults, supporting the compensation hypothesis.

Another limitation is that we collected structural data only in a sub-sample of younger adults (due to time constraints). Hence, we cannot fully exclude that GMV reductions in older adults appear different in case of the full sample of younger adults examined in our study.

## Author Contributions

LM, VD, and MS-A designed the study. LM, MS-A and AS recorded the data. LM and MS-A analyzed the data. LM wrote the paper. VD, AS, and MS-A read the paper and provided critical feedback.

## Conflict of Interest Statement

The authors declare that the research was conducted in the absence of any commercial or financial relationships that could be construed as a potential conflict of interest. The handling Editor declared a shared affiliation, though no other collaboration, with one of the authors LM.

## Footnotes

1 www.neurobs.com/presentation

2 http://www.fil.ion.ucl.ac.uk/spm/software/spm8/

## Supplementary Material

FIGURE S1 Box plots illustrating the beta-weights distribution during the non-cooperative task for three regions of interest (right PCG, left PCG, and right thalamus). *R*^2^-values represent within-group Pearson coefficients for beta-weight × age correlations, with (+) indicating a positive and (-) a negative correlation. ^∗^Indicates *p* < 0.05 (two-tailed unpaired *t*-test).

FIGURE S2 Illustration of the task × age correlation analyses across the whole sample. **(A)** Results for the cooperative movement task. **(B)** Results for the non-cooperative hand movement task. All results are shown on *p* < 0.05 (cluster-corrected).

## References

[B1] Ashburner J. (2007). A fast diffeomorphic image registration algorithm. *Neuroimage* 38 95–113. 10.1016/j.neuroimage.2007.07.007 17761438

[B2] Behzadi Y. Restom K. Liau J. Liu T. T. (2007). A component based noise correction method (CompCor) for BOLD and perfusion based fMRI. *Neuroimage* 37 90–101. 10.1016/j.neuroimage.2007.04.042 17560126 PMC2214855

[B3] Biswal B. Yetkin F. Z. Haughton V. M. Hyde J. S. (1995). Functional connectivity in the motor cortex of resting human brain using echo-planar MRI. *Magn. Reson. Med.* 34 537–541. 10.1002/mrm.1910340409 8524021

[B4] Blais M. Martin E. Albaret J. M. Tallet J. (2014). Preservation of perceptual integration improves temporal stability of bimanual coordination in the elderly: an evidence of age-related brain plasticity. *Behav. Brain Res.* 275 34–42. 10.1016/j.bbr.2014.08.043 25192640

[B5] Boujraf S. Summers P. Belahsen F. Prussmann K. Kollias S. (2009). Ultrafast bold fMRI using single-shot spin-echo echo planar imaging. *J. Med. Phys.* 34 37–42. 10.4103/0971-6203.48719 20126564 PMC2804146

[B6] Cabeza R. (2001). Cognitive neuroscience of aging: contributions of functional neuroimaging. *Scand. J. Psychol.* 42 277–286. 10.1111/1467-9450.00237 11501741

[B7] Cabeza R. Anderson N. D. Locantore J. K. Mcintosh A. R. (2002). Aging gracefully: compensatory brain activity in high-performing older adults. *Neuroimage* 17 1394–1402. 10.1006/nimg.2002.1280 12414279

[B8] Calautti C. Serrati C. Baron J. C. (2001). Effects of age on brain activation during auditory-cued thumb-to-index opposition: a positron emission tomography study. *Stroke* 32 139–146. 10.1161/01.STR.32.1.139 11136929

[B9] Coxon J. P. Goble D. J. Van Impe A. De Vos J. Wenderoth N. Swinnen S. P. (2010). Reduced basal ganglia function when elderly switch between coordinated movement patterns. *Cereb. Cortex* 20 2368–2379. 10.1093/cercor/bhp306 20080932

[B10] Damoiseaux J. S. Greicius M. D. (2009). Greater than the sum of its parts: a review of studies combining structural connectivity and resting-state functional connectivity. *Brain Struct. Funct.* 213 525–533. 10.1007/s00429-009-0208-6 19565262

[B11] Debaere F. Wenderoth N. Sunaert S. Van Hecke P. Swinnen S. P. (2003). Internal vs external generation of movements: differential neural pathways involved in bimanual coordination performed in the presence or absence of augmented visual feedback. *Neuroimage* 19 764–776. 10.1016/S1053-8119(03)00148-4 12880805

[B12] Dietz V. Macauda G. Schrafl-Altermatt M. Wirz M. Kloter E. Michels L. (2015). Neural coupling of cooperative hand movements: a reflex and fMRI study. *Cereb. Cortex* 25 948–958. 10.1093/cercor/bht285 24122137

[B13] Disbrow E. Roberts T. Poeppel D. Krubitzer L. (2001). Evidence for interhemispheric processing of inputs from the hands in human S2 and PV. *J. Neurophysiol.* 85 2236–2244. 10.1152/jn.2001.85.5.2236 11353038

[B14] Eudave L. Aznarez-Sanado M. Luis E. O. Martinez M. Fernandez-Seara M. A. Pastor M. A. (2016). Motor sequence learning in the elderly: differential activity patterns as a function of hand modality. *Brain Imaging Behav.* 10.1007/s11682-016-9569-7 27444732

[B15] Evans A. C. Marrett S. Neelin P. Collins L. Worsley K. Dai W. (1992). Anatomical mapping of functional activation in stereotactic coordinate space. *Neuroimage* 1 43–53. 10.1016/1053-8119(92)90006-9 9343556

[B16] Fontanini A. Katz D. B. (2008). Behavioral states, network states, and sensory response variability. *J. Neurophysiol.* 100 1160–1168. 10.1152/jn.90592.2008 18614753 PMC2544460

[B17] Fox M. D. Raichle M. E. (2007). Spontaneous fluctuations in brain activity observed with functional magnetic resonance imaging. *Nat. Rev. Neurosci.* 8 700–711. 10.1038/nrn2201 17704812

[B18] Fox M. D. Snyder A. Z. Zacks J. M. Raichle M. E. (2006). Coherent spontaneous activity accounts for trial-to-trial variability in human evoked brain responses. *Nat. Neurosci.* 9 23–25. 10.1038/nn1616 16341210

[B19] Friston K. J. Frith C. D. Turner R. Frackowiak R. S. (1995). Characterizing evoked hemodynamics with fMRI. *NeuroImage* 2 157–165. 10.1006/nimg.1995.1018 9343598

[B20] Goble D. J. Coxon J. P. Van Impe A. De Vos J. Wenderoth N. Swinnen S. P. (2010). The neural control of bimanual movements in the elderly: brain regions exhibiting age-related increases in activity, frequency-induced neural modulation, and task-specific compensatory recruitment. *Hum. Brain Mapp.* 31 1281–1295. 10.1002/hbm.20943 20082331 PMC6871108

[B21] Goble D. J. Coxon J. P. Van Impe A. Geurts M. Van Hecke W. Sunaert S. (2012). The neural basis of central proprioceptive processing in older versus younger adults: an important sensory role for right putamen. *Hum. Brain Mapp.* 33 895–908. 10.1002/hbm.21257 21432946 PMC6870471

[B22] Grady C. (2012). The cognitive neuroscience of ageing. *Nat. Rev. Neurosci.* 13 491–505. 10.1038/nrn3256 22714020 PMC3800175

[B23] Grady C. L. Maisog J. M. Horwitz B. Ungerleider L. G. Mentis M. J. Salerno J. A. (1994). Age-related changes in cortical blood flow activation during visual processing of faces and location. *J. Neurosci.* 14 1450–1462. 10.1523/JNEUROSCI.14-03-01450.1994 8126548 PMC6577560

[B24] Greicius M. D. Supekar K. Menon V. Dougherty R. F. (2009). Resting-state functional connectivity reflects structural connectivity in the default mode network. *Cereb. Cortex* 19 72–78. 10.1093/cercor/bhn059 18403396 PMC2605172

[B25] Hari R. Hanninen R. Makinen T. Jousmaki V. Forss N. Seppa M. (1998). Three hands: fragmentation of human bodily awareness. *Neurosci. Lett.* 240 131–134. 10.1016/S0304-3940(97)00945-2 9502221

[B26] Heitger M. H. Goble D. J. Dhollander T. Dupont P. Caeyenberghs K. Leemans A. (2013). Bimanual motor coordination in older adults is associated with increased functional brain connectivity–a graph-theoretical analysis. *PLoS One* 8:e62133. 10.1371/journal.pone.0062133 23637982 PMC3639273

[B27] Heuninckx S. Debaere F. Wenderoth N. Verschueren S. Swinnen S. P. (2004). Ipsilateral coordination deficits and central processing requirements associated with coordination as a function of aging. *J. Gerontol. B Psychol. Sci. Soc. Sci.* 59 225–232. 10.1093/geronb/59.5.P225 15358795

[B28] Heuninckx S. Wenderoth N. Debaere F. Peeters R. Swinnen S. P. (2005). Neural basis of aging: the penetration of cognition into action control. *J. Neurosci.* 25 6787–6796. 10.1523/JNEUROSCI.1263-05.2005 16033888 PMC6725362

[B29] Heuninckx S. Wenderoth N. Swinnen S. P. (2008). Systems neuroplasticity in the aging brain: recruiting additional neural resources for successful motor performance in elderly persons. *J. Neurosci.* 28 91–99. 10.1523/JNEUROSCI.3300-07.2008 18171926 PMC6671150

[B30] Honey C. J. Sporns O. Cammoun L. Gigandet X. Thiran J. P. Meuli R. (2009). Predicting human resting-state functional connectivity from structural connectivity. *Proc. Natl. Acad. Sci. U.S.A.* 106 2035–2040. 10.1073/pnas.0811168106 19188601 PMC2634800

[B31] Honey C. J. Thivierge J. P. Sporns O. (2010). Can structure predict function in the human brain? *Neuroimage* 52 766–776. 10.1016/j.neuroimage.2010.01.071 20116438

[B32] Hutchinson S. Kobayashi M. Horkan C. M. Pascual-Leone A. Alexander M. P. Schlaug G. (2002). Age-related differences in movement representation. *NeuroImage* 17 1720–1728. 10.1006/nimg.2002.1309 12498746

[B33] Kenet T. Bibitchkov D. Tsodyks M. Grinvald A. Arieli A. (2003). Spontaneously emerging cortical representations of visual attributes. *Nature* 425 954–956. 10.1038/nature02078 14586468

[B34] Kourtis D. De Saedeleer L. Vingerhoets G. (2014). Handedness consistency influences bimanual coordination: a behavioural and electrophysiological investigation. *Neuropsychologia* 58 81–87. 10.1016/j.neuropsychologia.2014.04.002 24732382

[B35] Labyt E. Cassim F. Szurhaj W. Bourriez J. L. Derambure P. (2006). Oscillatory cortical activity related to voluntary muscle relaxation: influence of normal aging. *Clin. Neurophysiol.* 117 1922–1930. 10.1016/j.clinph.2006.05.017 16887382

[B36] Labyt E. Szurhaj W. Bourriez J. L. Cassim F. Defebvre L. Destee A. (2003). Changes in oscillatory cortical activity related to a visuomotor task in young and elderly healthy subjects. *Clin. Neurophysiol.* 114 1153–1166. 10.1016/S1388-2457(03)00058-0 12804684

[B37] Labyt E. Szurhaj W. Bourriez J. L. Cassim F. Defebvre L. Destee A. (2004). Influence of aging on cortical activity associated with a visuo-motor task. *Neurobiol. Aging* 25 817–827. 10.1016/j.neurobiolaging.2003.08.010 15165706

[B38] Lin Y. Y. Forss N. (2002). Functional characterization of human second somatosensory cortex by magnetoencephalography. *Behav. Brain Res.* 135 141–145. 10.1016/S0166-4328(02)00143-2 12356444

[B39] Logan J. M. Sanders A. L. Snyder A. Z. Morris J. C. Buckner R. L. (2002). Under-recruitment and nonselective recruitment: dissociable neural mechanisms associated with aging. *Neuron* 33 827–840. 10.1016/S0896-6273(02)00612-8 11879658

[B40] Maes C. Gooijers J. Orban De Xivry J. J. Swinnen S. P. Boisgontier M. P. (2017). Two hands, one brain, and aging. *Neurosci. Biobehav. Rev.* 75 234–256. 10.1016/j.neubiorev.2017.01.052 28188888

[B41] Maldjian J. A. Laurienti P. J. Kraft R. A. Burdette J. H. (2003). An automated method for neuroanatomic and cytoarchitectonic atlas-based interrogation of fMRI data sets. *Neuroimage* 19 1233–1239. 10.1016/S1053-8119(03)00169-1 12880848

[B42] Mattay V. S. Fera F. Tessitore A. Hariri A. R. Das S. Callicott J. H. (2002). Neurophysiological correlates of age-related changes in human motor function. *Neurology* 58 630–635. 10.1212/WNL.58.4.630 11865144

[B43] Muller K. Bacht K. Prochnow D. Schramm S. Seitz R. J. (2013). Activation of thalamus in motor imagery results from gating by hypnosis. *Neuroimage* 66 361–367. 10.1016/j.neuroimage.2012.10.073 23128080

[B44] Murphy K. Birn R. M. Handwerker D. A. Jones T. B. Bandettini P. A. (2009). The impact of global signal regression on resting state correlations: are anti-correlated networks introduced? *Neuroimage* 44 893–905. 10.1016/j.neuroimage.2008.09.036 18976716 PMC2750906

[B45] Naccarato M. Calautti C. Jones P. S. Day D. J. Carpenter T. A. Baron J. C. (2006). Does healthy aging affect the hemispheric activation balance during paced index-to-thumb opposition task? An fMRI study. *Neuroimage* 32 1250–1256. 10.1016/j.neuroimage.2006.05.003 16806984

[B46] Nachev P. Kennard C. Husain M. (2008). Functional role of the supplementary and pre-supplementary motor areas. *Nat. Rev. Neurosci.* 9 856–869. 10.1038/nrn2478 18843271

[B47] Norman-Haignere S. V. Mccarthy G. Chun M. M. Turk-Browne N. B. (2012). Category-selective background connectivity in ventral visual cortex. *Cereb. Cortex* 22 391–402. 10.1093/cercor/bhr118 21670097 PMC3256407

[B48] Oldfield R. C. (1971). The assessment and analysis of handedness: the edinburgh inventory. *Neuropsychologia* 9 97–113. 10.1016/0028-3932(71)90067-4 5146491

[B49] Pan Z. Van Gemmert A. W. A. (2018). The control of amplitude and direction in a bimanual coordination task. *Hum. Mov. Sci.* 10.1016/j.humov.2018.03.014 [Epub ahead of print]. 29605439

[B50] Park D. C. Polk T. A. Park R. Minear M. Savage A. Smith M. R. (2004). Aging reduces neural specialization in ventral visual cortex. *Proc. Natl. Acad. Sci. U.S.A.* 101 13091–13095. 10.1073/pnas.0405148101 15322270 PMC516469

[B51] Power J. D. Barnes K. A. Snyder A. Z. Schlaggar B. L. Petersen S. E. (2012). Spurious but systematic correlations in functional connectivity MRI networks arise from subject motion. *Neuroimage* 59 2142–2154. 10.1016/j.neuroimage.2011.10.018 22019881 PMC3254728

[B52] Reuter-Lorenz P. A. Cappell K. A. (2008). Neurocognitive aging and the compensation hypothesis. *Curr. Dir. Psychol. Sci.* 17 177–182. 10.1111/j.1467-8721.2008.00570.x

[B53] Reuter-Lorenz P. A. Park D. C. (2014). How does it STAC up? Revisiting the scaffolding theory of aging and cognition. *Neuropsychol. Rev.* 24 355–370. 10.1007/s11065-014-9270-9 25143069 PMC4150993

[B54] Riecker A. Groschel K. Ackermann H. Steinbrink C. Witte O. Kastrup A. (2006). Functional significance of age-related differences in motor activation patterns. *Neuroimage* 32 1345–1354. 10.1016/j.neuroimage.2006.05.021 16798017

[B55] Roland P. E. Larsen B. Lassen N. A. Skinhoj E. (1980a). Supplementary motor area and other cortical areas in organization of voluntary movements in man. *J. Neurophysiol.* 43 118–136. 7351547 10.1152/jn.1980.43.1.118

[B56] Roland P. E. Skinhoj E. Lassen N. A. Larsen B. (1980b). Different cortical areas in man in organization of voluntary movements in extrapersonal space. *J. Neurophysiol.* 43 137–150. 10.1152/jn.1980.43.1.137 7351548

[B57] Rossiter H. E. Davis E. M. Clark E. V. Boudrias M. H. Ward N. S. (2014). Beta oscillations reflect changes in motor cortex inhibition in healthy ageing. *Neuroimage* 91 360–365. 10.1016/j.neuroimage.2014.01.012 24440529 PMC3988925

[B58] Sadaghiani S. Scheeringa R. Lehongre K. Morillon B. Giraud A. L. Kleinschmidt A. (2010). Intrinsic connectivity networks, alpha oscillations, and tonic alertness: a simultaneous electroencephalography/functional magnetic resonance imaging study. *J. Neurosci.* 30 10243–10250. 10.1523/JNEUROSCI.1004-10.2010 20668207 PMC6633365

[B59] Sala-Llonch R. Bartres-Faz D. Junque C. (2015). Reorganization of brain networks in aging: a review of functional connectivity studies. *Front. Psychol.* 6:663. 10.3389/fpsyg.2015.00663 26052298 PMC4439539

[B60] Schmiedt-Fehr C. Mathes B. Kedilaya S. Krauss J. Basar-Eroglu C. (2016). Aging differentially affects alpha and beta sensorimotor rhythms in a go/nogo task. *Clin. Neurophysiol.* 127 3234–3242. 10.1016/j.clinph.2016.07.008 27522489

[B61] Schneider-Garces N. J. Gordon B. A. Brumback-Peltz C. R. Shin E. Lee Y. Sutton B. P. (2010). Span, CRUNCH, and beyond: working memory capacity and the aging brain. *J. Cogn. Neurosci.* 22 655–669. 10.1162/jocn.2009.21230 19320550 PMC3666347

[B62] Schrafl-Altermatt M. Dietz V. (2014). Task-specific role of ipsilateral pathways: somatosensory evoked potentials during cooperative hand movements. *Neuroreport* 25 1429–1432. 10.1097/WNR.0000000000000285 25340563

[B63] Schrafl-Altermatt M. Dietz V. (2016a). Cooperative hand movements in stroke patients: neural reorganization. *Clin. Neurophysiol.* 127 748–754. 10.1016/j.clinph.2015.07.004 26275809

[B64] Schrafl-Altermatt M. Dietz V. (2016b). Neural coupling during cooperative hand movements after stroke: Role of ipsilateral afference. *Ann. Clin. Transl. Neurol.* 3 884–888. 10.1002/acn3.363 27844034 PMC5099534

[B65] Sepulcre J. Liu H. Talukdar T. Martincorena I. Yeo B. T. Buckner R. L. (2010). The organization of local and distant functional connectivity in the human brain. *PLoS Comput. Biol.* 10:e1000808. 10.1371/journal.pcbi.1000808 20548945 PMC2883589

[B66] Slotnick S. D. Moo L. R. Segal J. B. Hart J. Jr. (2003). Distinct prefrontal cortex activity associated with item memory and source memory for visual shapes. *Brain Res. Cogn. Brain Res.* 17 75–82. 10.1016/S0926-6410(03)00082-X 12763194

[B67] Tamè L. Pavani F. Braun C. Salemme R. Farnè A. Reilly K. T. (2015a). Somatotopy and temporal dynamics of sensorimotor interactions: evidence from double afferent inhibition. *Eur. J. Neurosci.* 41 1459–1465. 10.1111/ejn.12890 25879687

[B68] Tamè L. Pavani F. Papadelis C. Farnè A. Braun C. (2015b). Early integration of bilateral touch in the primary somatosensory cortex. *Hum. Brain Mapp.* 36 1506–1523. 10.1002/hbm.22719 25514844 PMC6869154

[B69] Vallesi A. Stuss D. T. (2010). Excessive sub-threshold motor preparation for non-target stimuli in normal aging. *Neuroimage* 50 1251–1257. 10.1016/j.neuroimage.2010.01.022 20079449

[B70] Van Impe A. Coxon J. P. Goble D. J. Wenderoth N. Swinnen S. P. (2009). Ipsilateral coordination at preferred rate: effects of age, body side and task complexity. *Neuroimage* 47 1854–1862. 10.1016/j.neuroimage.2009.06.027 19539766

[B71] Van Impe A. Coxon J. P. Goble D. J. Wenderoth N. Swinnen S. P. (2011). Age-related changes in brain activation underlying single- and dual-task performance: Visuomanual drawing and mental arithmetic. *Neuropsychologia* 49 2400–2409. 10.1016/j.neuropsychologia.2011.04.016 21536055

[B72] Vecchio F. Miraglia F. Bramanti P. Rossini P. M. (2014). Human brain networks in physiological aging: a graph theoretical analysis of cortical connectivity from EEG data. *J. Alzheimers Dis.* 41 1239–1249. 10.3233/JAD-140090 24820018

[B73] Ward N. S. (2006). Compensatory mechanisms in the aging motor system. *Ageing Res. Rev.* 5 239–254. 10.1016/j.arr.2006.04.003 16905372

[B74] Ward N. S. Frackowiak R. S. (2003). Age-related changes in the neural correlates of motor performance. *Brain* 126 873–888. 10.1093/brain/awg071 12615645 PMC3717766

[B75] Wenderoth N. Debaere F. Sunaert S. Swinnen S. P. (2005). Spatial interference during bimanual coordination: differential brain networks associated with control of movement amplitude and direction. *Hum. Brain Mapp.* 26 286–300. 10.1002/hbm.20151 15965999 PMC6871760

[B76] Whitfield-Gabrieli S. Nieto-Castanon A. (2012). *Conn*: a functional connectivity toolbox for correlated and anticorrelated brain networks. *Brain Connect.* 2 125–141. 10.1089/brain.2012.0073 22642651

[B77] Wu T. Hallett M. (2005). The influence of normal human ageing on automatic movements. *J. Physiol.* 562 605–615. 10.1113/jphysiol.2004.076042 15513939 PMC1665504

[B78] Zapparoli L. Saetta G. De Santis C. Gandola M. Zerbi A. Banfi G. (2016). When I am (almost) 64: the effect of normal ageing on implicit motor imagery in young elderlies. *Behav. Brain Res.* 303 137–151. 10.1016/j.bbr.2016.01.058 26851363

