# Supplementary figures and images for: Neuroplastic Changes in Older Adults Performing Cooperative Hand Movements
Source: Front Hum Neurosci. 2018 Dec 13;12:488. doi: 10.3389/fnhum.2018.00488 (PMC6300783; doi:10.3389/fnhum.2018.00488)

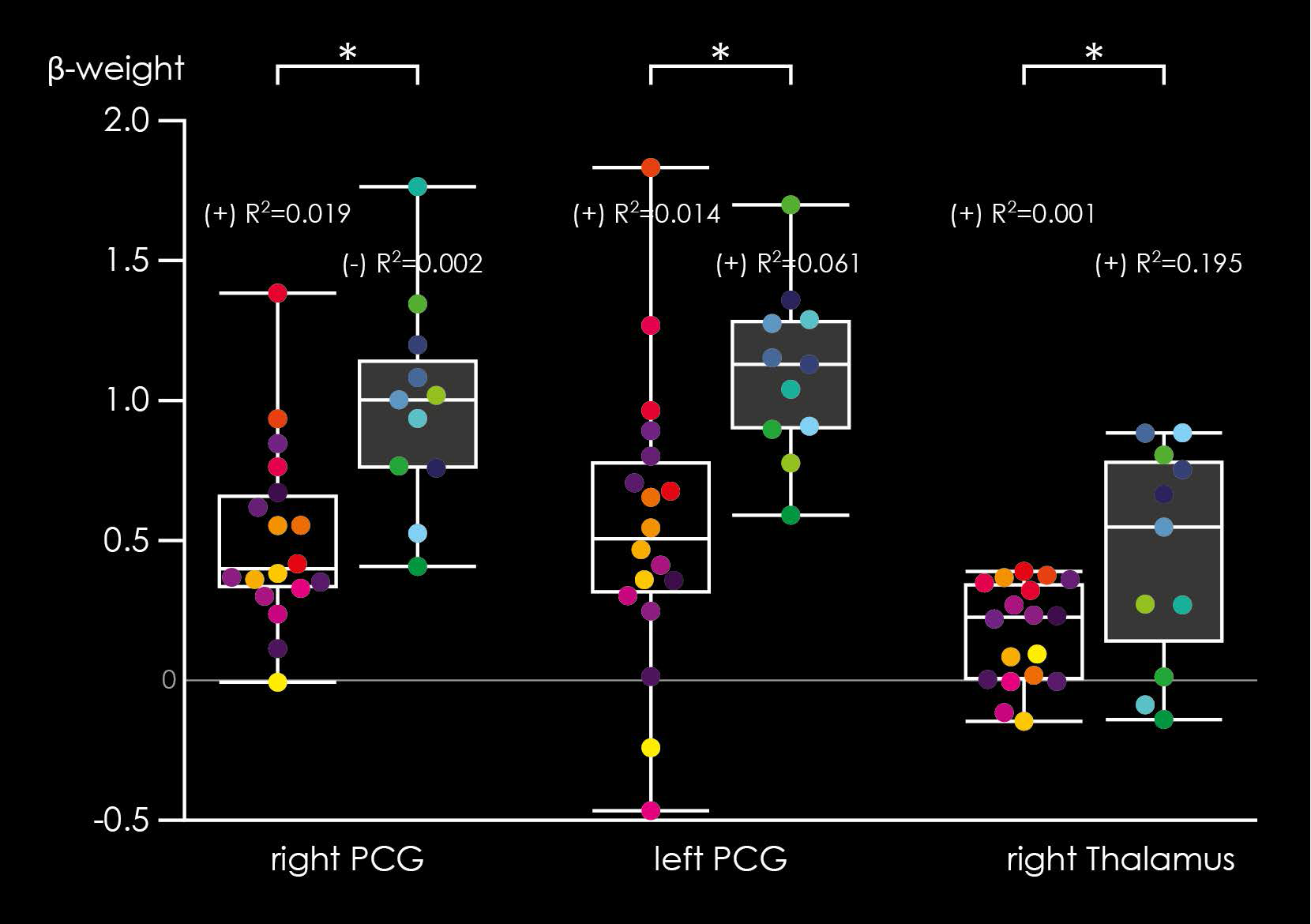

Supplement: FIGURE S1 — Box plots illustrating the beta-weights distribution during the non-cooperative task for three regions of interest (right PCG, left PCG, and right thalamus). R2-values represent within-group Pearson coefficients for beta-weight × age correlations, with (+) indicating a positive and (-) a negative correlation. ∗Indicates p < 0.05 (two-tailed unpaired t-test). [file Image_1.TIF]

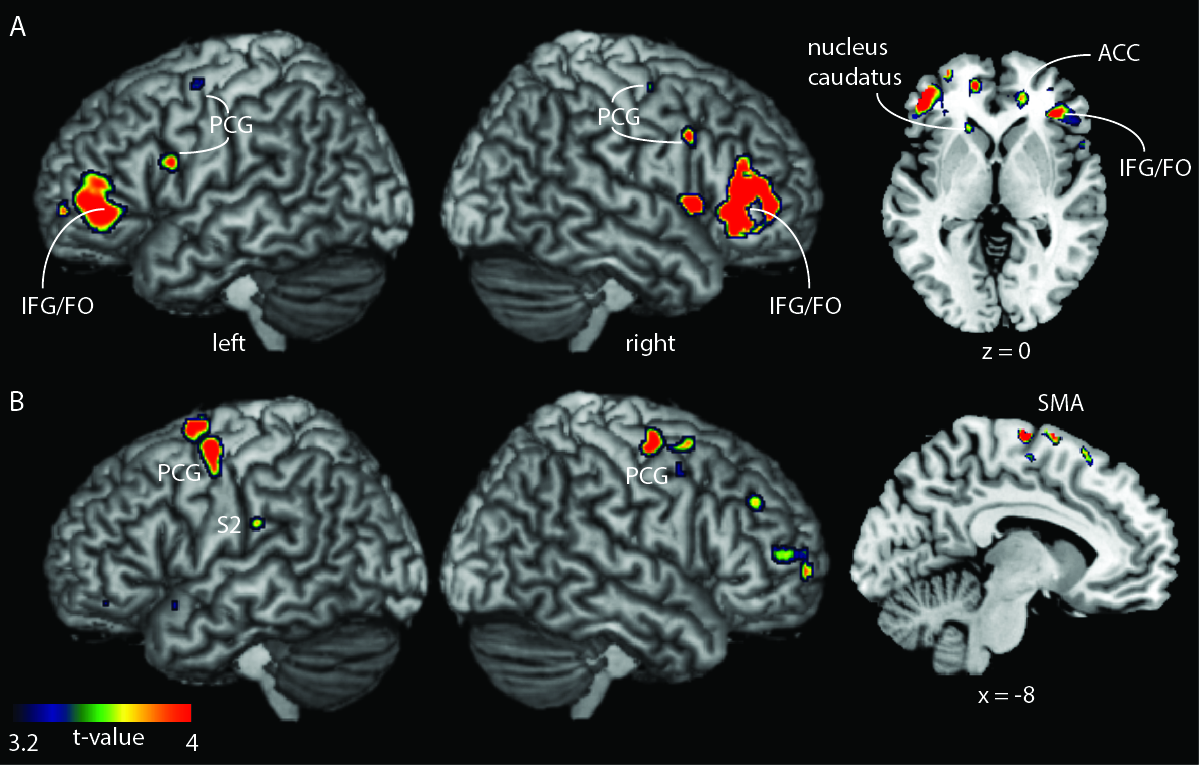

Supplement: FIGURE S2 — Illustration of the task × age correlation analyses across the whole sample. (A) Results for the cooperative movement task. (B) Results for the non-cooperative hand movement task. All results are shown on p < 0.05 (cluster-corrected). [file Image_2.TIF]
